# A Case Series and Review of Febrile-Infection Related Epilepsy Syndrome (FIRES)

**DOI:** 10.3390/children12040485

**Published:** 2025-04-10

**Authors:** Tahnee Spoden, Alice Hoftman, Nanci Rascoff, Deborah McCurdy

**Affiliations:** Division of Allergy/Immunology/Rheumatology, University of California, Los Angeles, CA 90095, USA; ahoftman@mednet.ucla.edu (A.H.);

**Keywords:** new onset refractory status epilepticus (NORSE), febrile infection-related epilepsy syndrome (FIRES), cryptogenic NORSE, cryptogenic FIRES, children, cytokines, systemic immunotherapy, JAK inhibitor

## Abstract

Background: FIRES is a rare and catastrophic presentation of a de novo refractory status epilepticus (RSE) in healthy individuals following mild febrile illness. It carries a high burden of morbidity and an estimated mortality of 12% in children. In over half of patients, an underlying cause is not discovered (cryptogenic FIRES). The theory that post-infectious inflammation promotes aberrant neuronal excitation has led to the use of immunomodulatory therapies as treatment for FIRES. High-dose glucocorticoids and intravenous immunoglobulin (IVIG) are used as first-line therapies but are ineffective in most cases. A comprehensive initial evaluation is critical in directing second-line therapies; however, an autoimmune and inflammatory workup is seldom completed prior to treatment. Despite recent trends toward using cytokine-directed therapies, outcomes remain poor. Methods: This single-institution retrospective case series describes three cases of FIRES in similarly aged children. Each patient experienced super-refractory status epilepticus (SRSE) resistant to first-line systemic immunotherapy (SIT). The novel use of baricitinib, a non-selective JAK inhibitor, proved effective for one patient, while IL-1 and IL-6 inhibition were effective in the other two. All patients suffered moderate-to-severe neurologic and cognitive impairment at the time of discharge. Conclusions: FIRES is a poorly understood catastrophic presentation of refractory status epilepticus (RSE) requiring a multimodal approach to treatment. Cytokine profiling can be helpful in identifying cryptogenic cases from those with an underlying cause if conducted early in the clinical course. The early use of second-line immunomodulatory therapies may aid in decreasing neuroinflammation and improve outcomes.

## 1. Introduction

Febrile infection-related epilepsy syndrome (FIRES) is a rare and catastrophic presentation of status epilepticus (SE) affecting approximately one in one million children with an estimated mortality rate of 12%. Of those that survive, 90% suffer permanent neurocognitive deficits and chronic epilepsy [1,2]. This phenomenon occurs in healthy individuals within 2 weeks of a mild febrile illness. FIRES is classified as a subcategory of new onset refractory status epilepticus (NORSE) (see Table 1) where SE without an identifiable cause is refractory to two or more anti-epileptic medications [2,3,4]. In roughly half of patients, an underlying cause is never found, and this is termed cryptogenic NORSE/FIRES (c-NORSE/c-FIRES).

Most pediatric patients diagnosed with NORSE have a preceding febrile illness, thus meeting criteria for FIRES [2,3,5,6,7,8,9]. Autoimmune encephalitis and paraneoplastic syndromes are more common in adults and often present with focal neurologic findings or psychiatric symptoms [5,10]. In a small, but not negligible, number of pediatric patients, a genetic or metabolic etiology is discovered [2,8,11]. Several studies have demonstrated that FIRES in the pediatric population is more likely to be cryptogenic as compared to adults [12]. A retrospective study of 71 patients aimed to identify characteristics that could differentiate c-NORSE from RSE with an identifiable cause [8]. Patients with c-NORSE more frequently had prodromal fever, required mechanical ventilatory support, and had symmetric abnormalities on MRI. Those with a genetic cause were younger in age and less likely to progress to SRSE. Cases where an etiology was discovered were associated with CSF oligoclonal bands, psychoactive behavioral alterations, and involuntary movements [8]. The early identification of some of these features can help direct investigation and treatment.

The clinical progression in NORSE is biphasic. In the acute phase, children can have hundreds of seizures a day and frequently require multiple anti-epileptic drugs (AEDs) and anesthetic medications to suppress seizure activity. The acute phase can last days to months. After RSE has resolved, the chronic phase results in neurocognitive deficits and drug-resistant epilepsy in most cryptogenic cases [1,5,10,13]. Cryptogenic cases have proven to be more resistant to anti-seizure medications with higher morbidity and mortality [2]. It is not known what causes FIRES or what might predispose children to developing FIRES. A widely accepted theory is that unregulated systemic inflammation leads to inflammation within the CNS. Pro-inflammatory cytokines trigger aberrant neuroexcitation, leading to drug-resistant seizures [14]. Anti-inflammatory and immunomodulatory therapies have become the standard of care in the treatment of NORSE/FIRES with variable results [2]. Advances in cytokine analysis has led to the use of cytokine-directed therapies such as anakinra (IL-1 inhibition) and tocilizumab (IL-6 inhibition) [2,14]. JAK-STAT pathways have been implicated in various forms of epilepsy and are another potential target of treatment [15]. Despite multiple JAK inhibitors (JAKi) currently in use for various inflammatory diseases, the use in FIRES is limited to a few case reports [16,17]. Research remains lacking on which therapies would provide the most benefit in cryptogenic cases.

**Table 1:** Definitions.

Status Epilepticus (SE)	Generalized seizure lasting longer than 5 min Focal seizure with impaired consciousness lasting 10 min Absence seizure lasting 10–15 min
Refractory Status Epilepticus (RSE)	Status epilepticus persists despite administration of 2 or more parenteral anti-seizure medications
Super Refractory Status epilepticus (SRSE)	Status epilepticus persists at least 24 h after onset of anesthesia without interruption of appropriate anesthesia, recurring while on appropriate anesthesia or recurring after withdrawal of anesthesia, requiring re-introduction of anesthetic
New Onset Refractory Status Epilepticus (NORSE)	The clinical presentation of new onset refractory status epilepticus, in a patient without history of epilepsy, that has no clear acute or active structural, toxic, or metabolic cause
Febrile Infection-Related Epilepsy Syndrome (FIRES)	A subcategory of NORSE that requires a preceding febrile infection with fever occurring between 2 weeks and 24 h prior to the onset of RSE. Fever does not need to be present at onset of SE

Sources for above definitions [2,3,4,14].

The high mortality and morbidity rates associated with NORSE/FIRES necessitates patients be treated in a tertiary center by a multidisciplinary team of specialists familiar with neuroinflammatory disorders. The rarity of this condition and paucity of pediatric providers with knowledge in the immunopathology associated with FIRES leads to delays in treatment and a less robust use of immunomodulatory therapies. While there are increasing numbers of case reports discussing the use of second-line immunomodulatory therapies in the treatment of FIRES, the majority of studies are limited to first-line treatment with IVIG, IV glucocorticoids, and, in some cases, rituximab. This case series highlights three school-age children who were transferred to our tertiary center with FIRES who progressed to SRSE. It illustrates the clinical progression of FIRES with an emphasis on immunodiagnostics and immunomodulatory treatments. To our knowledge, this is the only case report utilizing baricitinib (JAKi) in the treatment of FIRES.

## 2. Materials and Methods

This case series presents the clinical features, laboratory and imaging data, and treatments for three children who were transferred to the University of California, Los Angeles, Mattel Children’s Hospital from 2022 to 2024 for a higher level of care. Although, the data collection was compiled retrospectively the patients were cared for by each of the authors. There was no inclusion or exclusion criteria since the patients presented sequentially, and, to the author’s knowledge, there were no other patients with NORSE or FIRES admitted during this time. Each patient was analyzed for infectious, autoimmune, and metabolic causes but was felt to fulfill most closely the description of FIRES. FIRES was defined as new onset refractory status epilepticus in an individual who had a known fever or infection within 14 days and 24 h prior to the onset of seizure [2]. Patients with known epilepsy, metabolic, structural, infectious, or other toxicities leading to RSE discovered within 72 h of seizure onset were excluded from the definition. Information pertaining to clinical presentation, laboratory results, imaging results, EEG findings, treatment, complications, and neurologic outcomes at time of discharge were compared, as shown in Table 2, Table 3 and Table 4. Since there are only 3 patients presented, there is no statistical analysis, but 2 of the patients responded to therapies as discussed [2].

## 3. Results

### 3.1. Case Presentation

#### 3.1.1. Case 1

A 10-year-old male with no significant past medical history presented to an outside emergency department (ED) after he developed lip smacking and mouth twitching followed by a generalized tonic–clonic seizure lasting 5–7 min. Six days prior, he experienced a headache and low-grade fever of 100.3 F and was diagnosed with a viral syndrome by his local physician. While in the ED, he experienced an episode of left lateral gaze and left-hand tensing and was loaded with levetiracetam and transferred to a local PICU. Initial evaluation in the ED revealed a normal head CT and brain MRI. Labs were significant for mildly elevated CRP. CSF studies were normal except for mildly elevated glucose. Infectious workup was negative. Anti-NMDA receptor ab was later found to be negative. EEG was performed showing status epilepticus with focal seizure origination in the right temporoparietooccipital area. He was then sedated with midazolam and intubated. Systemic immunotherapy (SIT) was initiated on day three with intravenous immunoglobulin (IVIG) and IV methylprednisolone (dose and duration in Table 3). He remained in SRSE and was placed in a pentobarbital coma. SRSE persisted prompting transfer to a tertiary center for higher level of care. Upon transfer, it was noted that his medications were dosed based on ideal weight, doses were increased, and he achieved burst suppression for a short time. Ketamine was then started as well as additional AEDs (noted below in Table 2) to achieve burst suppression. Repeat MRI showed bilateral restricted diffusion of the hippocampus. Pediatric rheumatology was consulted on hospital day 8. Repeat CSF and serum studies were obtained as well as imaging to evaluate for an autoimmune or paraneoplastic process. Paraneoplastic evaluation with CT chest/abdomen/pelvis as well as the testicular ultrasound were negative. CSF studies again showed a normal cell count and protein count. Myelin basic protein was mildly elevated in the CSF; however, the IgG synthesis rate and oligoclonal bands were negative. Serum cytokines showed mild elevations in IL-6 and IL-8 on day 9 of hospitalization after he had received steroids, IVIG, and was started on anakinra. CSF cytokines were sent to Cincinnati Children’s laboratory much later in the hospital course and were normal at that time. Autoantibody studies were grossly negative aside from a positive anti-TPO antibody and anti-thyroglobulin antibody, which both normalized on subsequent testing, suggesting false positivity post-IVIG. He was given a second course of IV methylprednisolone. His autoimmune encephalitis workup resulted with a positive GAD65 antibody in the serum and a mildly positive antibody in the CSF, prompting a five-day course of plasmapheresis. A ketogenic diet was started on hospital day 12; however, ketosis was difficult to achieve. After a lack of improvement in his EEG findings following increased doses of anakinra, tocilizumab (IL-6 receptor antagonist) was started. His serum IL-6 levels were monitored periodically through ARUP and Cincinnati laboratories prior to starting tocilizumab and after its initiation. IL-6 levels rose just prior to starting tocilizumab and remained elevated with monitoring. After his second dose of tocilizumab, there was some improvement in his EEG activity, and pentobarbital was successfully weaned. Seizures returned with an attempted wean of ketamine, and he was given a third dose of tocilizumab. He continued to have breakthrough seizures. He received a third pulse of methylprednisolone on hospital day 48 with no improvement in ability to wean off anesthetic agents. He struggled with rising transaminases, likely due to the large pharmacologic burden from AEDs and antibiotics needed for recurrent infections. His seizure burden increased despite increased doses of ketamine. He was then started on baricitinib (JAKi), and clinical and subclinical seizure activity resolved within 48 h. Cytokine monitoring showed a steady decline in serum IL-6 after starting baricitinib. His baricitinib dose was increased following some breakthrough seizure activity but was then subsequently decreased due to recurrent episodes of pneumonia. He was weaned off ketamine on hospital day 83, at which time he began to have increased alertness and interaction. He was discharged to inpatient rehabilitation and maintained on baricitinib for nearly 12 months. Upon follow up, he remains on several AEDs. He has breakthrough seizures upon weaning AEDs, which eventually resolve without increased therapy. He participates in home schooling and home-based occupational and physical therapies, showing gradual improvements in his motor and cognitive function. He remains in a state of moderate disability.

#### 3.1.2. Case 2

A 6-year-old male with no significant past medical history was brought to his local ED after being found unresponsive by his mother with periorbital cyanosis and left-sided facial twitch. Six days prior to presentation, he had a fever lasting four days, fatigue, and loss of appetite. He had defervesced for two days and was in his usual state of health until he awoke that morning at 5:00 am with chest pain. He went back to bed and was discovered by his mother three hours later unconscious and seizing. On initial evaluation in the ED, he was afebrile and unresponsive. He was given Ativan, levetiracetam, and valproic acid. Initial laboratory workup was notable for leukopenia, anemia, and a normal CSF analysis. A CT scan of the brain was performed and was normal. He was transferred to a local children’s hospital and placed on EEG, which revealed that he was in subclinical SE. Repeat neuroimaging with MRI was significant for hyperintensities in the bilateral basal ganglia. Additional AEDs and sedative medications were given (see Table 2). Repeat CSF studies were normal as was his autoimmune encephalitis antibody panel, which resulted later. Infectious studies were unrevealing. On day two of seizures, he was started on IVIG and high-dose IV methylprednisolone (dose and duration in Table 3). After 10 days of hospitalization and treatment with multiple AEDs, a pentobarbital coma, and first-line SIT, he remained in SRSE. Plasmapheresis was initiated as well as a ketogenic diet, although he had difficulty achieving ketosis. Second-line SIT with subcutaneous anakinra was started on day 19 of hospitalization due to little improvement in clinical status. Attempts to wean sedative medications were met with the recurrence of seizures. He was transferred to a higher level of care on hospital day 37. During transport, all his sedative infusions were stopped, and he arrived in the ED in clinical SE. Burst suppression was achieved after re-titrating multiple AEDs and initiating multiple IV sedative medications. Repeat CSF studies showed 2000 RBCs, elevated protein, an elevated IgG synthesis rate, and myelin basic protein consistent with CNS inflammation and injury. Repeat MRI showed findings of hemorrhagic posterior reversible encephalopathy syndrome (PRES), likely from prolonged status epilepticus. Anakinra was resumed and given intravenously. He had recurrence of seizures a week later and serum cytokines revealed elevated soluble IL-2R. He was given another three-day course of high dose methylprednisolone, and anesthetics were increased. His seizure burden improved, and sedative medications were able to be successfully weaned without the recurrence of SE. He continued to have choreoathetoid movements throughout admission that did not correlate with EEG findings. These improved with the addition of diazepam. The evidence of hepatic injury prompted a liver biopsy with findings consistent with drug-induced hepatitis. Felbamate was discontinued, and phenobarbital was weaned. He maintained good seizure control during the month leading to transfer to inpatient rehabilitation. Upon transfer to acute rehabilitation, he continued to remain in a state of severe neurocognitive disability on ventilatory support. Anakinra was continued upon discharge.

#### 3.1.3. Case 3

An 8-year-old girl with no significant past medical history presented to her local ED in a state of aggressive demeanor following a witnessed generalized tonic–clonic seizure lasting one minute at home. Six days prior, she experienced a mild sore throat and abdominal pain followed by a fever of 104 F. Her fever resolved after 3 days, and she was able to return to school. While at school, she began to experience extreme fatigue and headache and was picked up early. Upon arriving home, she complained of right arm pain and right eye pain followed by her first seizure. She was afebrile when arriving at the ED with normal inflammatory markers, CBCs, and metabolic panel. CSF studies were notable for mildly elevated glucose and a normal WBC count with a neutrophil predominance on the differential. MRI of the brain showed mild FLAIR hyperintensities in the bilateral sulci. She experienced a recurrence of SE while being evaluated in the ED, which progressed to RSE. EEG findings noted that the seizures were generalized and originated in the left hemisphere. Her RSE progressed to SRSE, and first-line SIT with IV methylprednisolone and IVIG were initiated on day 2 of hospitalization (doses and duration of SIT in Table 3). She was transferred to a tertiary center for a higher level of care, and burst suppression was eventually achieved after the addition of pentobarbital and ketamine. Despite the completion of first-line SIT, she remained in SRSE. Anakinra was started on day 7 of hospitalization for a second-line SIT. Workup for an autoimmune process resulted with a positive SSA concerning for possible Sjogren’s encephalitis. Autoimmune workup was not performed until after she received IVIG; however, the SSA titer was higher than what would be expected as a false positive post-IVIG. She was started on plasmapheresis followed by rituximab, an anti-CD-20 directed biologic, a second-line treatment for autoimmune encephalitis. Serum cytokines were obtained showing elevated levels of soluble IL-2R as well as cytokines involved in both Th2 and Th17 cellular responses (see Table 4). Given the lack of significant improvement in her SRSE after 2 weeks of therapy, anakinra was discontinued, and she was started on tocilizumab. Sedative medications were able to be weaned two days later, and all sedation was discontinued two weeks later. She developed a complication of severe macroglossia, which started just prior to starting tocilizumab. A definitive cause of her macroglossia was not found, and her tongue slowly returned to its normal size over the next two months. Complications of nephrolithiasis led to a mild increase in self-resolving seizures, which improved after the renal stones were passed. There was a brief period of bilateral wrist and ankle swelling with overlying warmth and restricted range of movement concerning for arthritis. This improved over the course of her hospitalization. On day 40 of hospitalization, her level of consciousness began to improve with spontaneous eye opening. One month later, she was following commands and attempting to speak. Prior to discharge, she was alert and interactive and able to articulate needs. She was transferred to acute inpatient rehabilitation. Neurocognitive deficits remaining two months after discharge included short term memory loss, difficulty with concentration, and behavioral outbursts. She was able to enroll in home schooling and retained her ability to read, write, and perform grade level mathematics. Persistent motor weakness, predominantly in her lower extremities, was addressed with intensive physical therapy.

## 4. Discussion

### 4.1. Proposed Pathogenesis

Post-infectious immune activation plays a key role in the development of FIRES. Disruption of the blood–brain barrier in the setting of systemic inflammation triggers neural excitation leading to the development of seizures [18,19,20,21,22]. Cytokines readily cross the blood–brain barrier (BBB), leading to the activation of microglia and astrocytes that produce inflammatory cytokines locally in the CNS. In animal models, rats with inflammatory bowel disease showed elevated proinflammatory cytokines in the CNS that mirrored those found in the periphery. This mirrored inflammation led to the proliferation of microglia. These rats showed increased susceptibility to seizures during states of inflammation, and the susceptibility was reduced with TNF-α inhibition [23]. Another proposed mechanism of CNS activation is through cytokine-induced upregulation of adhesion molecules on the internal vessels of the brain. This facilitates the increased extravasation of inflammatory cells across the BBB. Once activated, microglia and astrocytes produce proinflammatory cytokines such as IL-1β and TNF-α. IL-1β has been shown to be a pro-convulsant cytokine by activating the NMDA receptor and inhibiting glutamate reuptake [19,20,22]. In cases of FIRES, the low-grade systemic inflammatory prodrome in the days to weeks prior to SE is thought to activate inflammatory cells in the CNS through the above mechanisms.

Inflammatory profiles in FIRES patients differ from other CNS disorders. Cytokine and chemokine profiles in children with FIRES show an elevation of Th1 specific cytokines (IL-2, TNF, INF-γ) and chemokines when compared to children with other forms of epilepsy or encephalopathy [24]. Despite the wealth of evidence for the role of IL-1β in neuroexcitation, IL-1β was not found to be elevated in FIRES patients in two different studies; however, cytokines involved in the innate immune response (IL-6, IL-8, and CCL2) were elevated [12,24,25]. While FIRES is classified as a subset of NORSE, two systematic reviews found that the cytokine and chemokine profiles in NORSE involved both the innate and adaptive immune systems, while profiles in FIRES patients lacked the evidence of an adaptive immune response [5,21]. Brain biopsies of patients with FIRES and cryptogenic NORSE show generalized neuronal cell loss and reactive gliosis. Evidence of the activation of the adaptive or humoral immune system is often absent [5,21,26]. The level of innate immune activation in FIRES patients indicates that CNS inflammation likely contributes to seizure onset and severity and is not merely a consequence of the seizure itself.

It is unclear what may predispose an individual to FIRES. The responses of viral and bacterial sensing toll-like receptors (TLR3, TLR4, TLR7/8 and TLR9) were found to be impaired in the peripheral mononuclear cells of five children with FIRES. It is thought that the decreased clearance of viral and bacterial debris during infection led to epitope cross-reactivity in neuronal components in the CNS. Additionally, these patients had reduced regulatory T-cells, which led to impaired downregulation of the inflammatory cascade [27]. While the proposed mechanism of autoimmune activation is contrary to the theory of FIRES being primarily an overactivation of the innate immune system, dysregulation of the immune system was demonstrated in this cohort.

Genetic alterations in the genes encoding IL-1Ra have also been found in pediatric patients with FIRES [21,28]. The IL-1Ra is important in the downregulation of IL-1 activity. Variable number tandem repeat polymorphisms in the IL1RN gene were found in 19 children with FIRES. Specifically, the haplotype *RN2* of *IL1RN,* which leads to decreased IL-1Ra production, carried a higher risk of encephalopathy [29]. A specific environmental or genetic predisposition has not yet been discovered. It is of note that two of our patients had a family history of juvenile idiopathic arthritis (JIA). It is likely a combination of factors including dysregulation of the immune system, BBB disruption, and a lowered seizure threshold in susceptible individuals.

### 4.2. Recommended Evaluation

In patients who present with clinical features of FIRES, as discussed above, a prompt and thorough workup with an early initiation of treatment leads to the best outcome. It is recommended to initiate first-line SIT no later than 72 h after the onset of RSE. First-line therapies include IVIG and high-dose steroids. If these therapies are initiated prior to a serological and inflammatory workup, the results may be less reliable and more difficult to interpret, as was seen in two of our patients. The international consensus recommendations for the management of NORSE outline the recommended workup, which includes evaluations for infectious, autoimmune, paraneoplastic, metabolic, and genetic causes [2,14]. Extra blood and CSF samples for storage and future analyses should also be obtained in case additional studies are needed after the initiation of SIT. CSF studies are important in differentiating c-NORSE from other etiologies. Given the time-sensitive nature and need for larger volumes of CSF in these patients, it is our recommendation that lumbar puncture be performed by an experienced clinician that is skilled in this procedure.

CSF analysis is a critical first step in the workup of new onset seizures. Ruling out infections can be lifesaving. In addition to helping to ruling out infection, additional studies can help differentiate c-NORSE from other causes. CSF white blood cell counts are typically normal in FIRES, as was seen in our cohort, or show a mild pleocytosis. Elevations in CSF protein are frequently seen in epilepsy [30]. CSF protein was elevated upon repeat analysis in one of our patients; however, there was also a very high RBC count with MRI findings showing hemorrhagic PRES, which could account for the elevated protein. CSF oligoclonal bands and the IgG synthesis rate indicates intrathecal IgG production that is independent of systemic inflammation. Oligoclonal bands are often seen in multiple sclerosis (MS) but can also be seen in other inflammatory processes such as autoimmune encephalitis or infection. Mirrored bands, bands seen in the CSF and serum, can indicate BBB dysfunction [31]. Elevated oligoclonal bands have been shown to be negatively associated with c-NORSE and, if present, may prompt the clinician to evaluate for another underlying cause [32]. An elevated IgG synthesis rate was seen as a later finding in one of our patients with c-NORSE, and none of our patients had elevated oligoclonal bands in the CSF. Other markers of neuronal damage, such as CNS myelin basic protein (MBP), can indicate demyelinating diseases such as MS or acute demyelinating encephalomyelitis (ADEM). MBP can also be present after CNS trauma and hydrocephalus in children and indicate CNS damage [33,34,35]. MBP was elevated upon repeat CSF analysis in two patients in our cohort; however, it was likely more of an indication of CNS damage due to SRSE rather than a primary demyelinating process given the lack of clinical and radiographical correlates. As specific serologic markers of autoimmune encephalitis can take weeks to result, a careful analysis of readily available CNS studies can help rule out other causes and dictate treatment.

While autoimmune causes of NORSE/FIRES are less frequently seen in children, a comprehensive autoimmune workup is important in all patients as it can direct treatment and prognosticate outcomes. The international consensus recommendations highlight testing to include anti-neuronal surface antigens, myelin oligodendrocyte glycoprotein (MOG), glutamic acid decarboxylase 65 (GAD65), anti-thyroid, paraneoplastic, and systemic lupus erythematosus (SLE) panels, anti-nuclear antibodies (ANAs), and antineutrophil cytoplasmic antibodies (ANCAs) as a standard workup [2,14]. Additionally, complete rheumatologic evaluation can include the addition of other autoantibodies involved in systemic processes that may affect the CNS. All patients in our cohort had autoantibody testing with one patient having a full autoimmune encephalitis and paraneoplastic panel sent prior to IVIG. The other had testing for specific antibodies such as anti-NMDA and MOG prior to IVIG but lacked a complete workup. The presence of autoantibodies at low titers can often be false positives. The question of their pathogenicity is complicated further after a patient receives IVIG. A significantly elevated anti-SSA antibody was detected in one of our patients, which prompted treatment with plasmapheresis and rituximab. It is unclear if this was a true positive SSA as it was drawn after IVIG. Plasmapheresis and rituximab can be effective in autoantibody mediated diseases; however, they are not without side effects, and risk vs. benefit hinges on the reliability of testing. This patient went on to receive both plasmapheresis and rituximab but required additional cytokine-directed therapies before seizures aborted.

In cases of c-FIRES, CSF cytokine and chemokine profiles are used for diagnostic and prognostic purposes. CSF cytokine profiles have been shown to differ in cases of NORSE/FIRES as compared to other CNS inflammatory disorders [12,19,23,24,25,29,36,37]. A retrospective study measuring intrathecal cytokines and chemokines in pediatric patients showed that children with FIRES had higher CNS cytokines associated with a Th1-mediated immune response (TNF-α, CXCL9, CXCL10, CXCL11) as well as CCL19, CXCL1, and IL-6 when compared to children with febrile status epilepticus or other non-inflammatory CNS disorders. Children with encephalitis had more of a diffuse clustering of B-cell and T-cell-associated cytokines [24]. Different cytokine profiles have also been shown to predict outcomes [38,39]. CSF cytokine analysis was not performed early in the clinical course in any of our patients and was only obtained in two patients during their entire clinical course. IL-6 was elevated in one patient at time of active RSE without elevation of other cytokines. CSF cytokine analysis was normal in another patient despite some seizure activity. The patient with the elevated IL-6 in the CSF did result with the poorest neurologic outcome at the time of discharge and had the worst seizure burden overall. In cryptogenic cases, cytokine-directed therapy has been reported to be effective when first and second-line treatments fail [17,40,41]. The lack of routine utilization of cytokine analysis as well as the difficulty in performing periodic CSF analysis in pediatric patients makes CSF cytokine profiling difficult in practice.

Serum cytokine analysis was performed periodically in all our patients. Serum IL-6 was elevated most often in all patients. Serum IL-6 levels did not seem to decrease with the use of tocilizumab in two of our patients and remained elevated despite good seizure control on tocilizumab in case 3. Serum IL-6 levels did begin to decrease after the initiation of baricitinib and correlated with seizure activity and overall improved neurologic status in one patient. Over time, serum cytokines may be less reflective of the initial underlying etiology as the patients frequently experience infections, procedures, or changes in medications, which may alter the cytokine profile. Provider variability also led to inconsistent serum cytokine monitoring intervals as well as laboratory preference for analysis, making it difficult to compare the three cases above. Consistencies in monitoring will help with future analysis.

Imaging is important in the initial workup of FIRES and should be performed within the first 48 h. It is recommended to obtain MRI with gadolinium contrast and consider MRA and MRV early in the clinical period. Gadolinium contrast can help identify blood–brain barrier disruption. In the acute phase of NORSE/FIRES, MRI is found to be normal in most patients. In one study, T1/FLAIR hyperintensities in the mesial temporal lobe, neocortical areas, or basal ganglia were seen in most patients [9]. Abnormalities in the temporal lobe followed by the basal ganglia encompassed most findings in a large literature review of children with FIRES [42]. The same review reported the most common findings in the chronic phase to be generalized brain atrophy. MRI findings in conjunction with the history of presentation can help differentiate between FIRES, ADEM, or limbic encephalitis. Limbic encephalitis will also show bilateral temporal lesions. However, the onset is more insidious, and a prodrome of memory impairment is common. Brain spectroscopy can be helpful in identifying findings consistent with inborn errors of metabolism and should be strongly considered in younger pediatric patients or when there is clinical suspicion [2]. While paraneoplastic causes are more common in adults, screening should be performed in all patients with a CT chest, abdomen, and pelvis. It is recommended to additionally obtain testicular and ovarian ultrasound as a part of malignancy screening as there is a strong association between teratomas and antibody-mediated encephalopathies [2].

EEG findings can help in the diagnosis of patients with NORSE/FIRES. One retrospective single-center study analyzed findings in children meeting criteria for FIRES and noted specific features common in most patients. These features included an initial lack of electrographic seizures with a gradual increase in activity leading up to SE. There was an extreme delta brush (EDB) pattern in the background followed by characteristic electrographic seizures arising from the frontal, temporal, or central areas and spreading [43]. Similar patterns of background delta slowing were seen in our cohort as well as focal onset leading to the generalized spreading of seizures.

Genetic evaluation should be pursued in most cases of NORSE and should be considered early in young patients [2]. Polymorphisms in the *SCN1A* and *SCN2A,* genes associated with Dravet syndrome and febrile seizures were found with statistical significance in patients with FIRES indicating that an undiagnosed seizure disorder could be the cause [29]. Another retrospective single-center study looking at 71 pediatric patients with NORSE found that a genetic cause could be attributed to 21.1% of patients. Undiagnosed metabolic disorders were the cause in 3% [8]. Other studies have additionally demonstrated that, despite the lack of typical features associated with metabolic and genetic disorders prior to FIRES, a significant number are identified [2,5]. Given the ease and relative affordability of genetic testing today, the benefit to testing in the pediatric population outweighs the risk. Whole exome sequencing was performed in two of our patients. There were VUS’s identified in both patients in genes that are known to be involved in axonal transport (KIF1A), CNS, cardiac potassium channel function (KCNH2), and associations with lateral temporal epilepsy (MICAL1) [44,45,46]. The patients’ specific mutations were not previously reported in the literature relating to epilepsy. Genetic studies are not routinely performed lending to a paucity of available data for comparison. The VUS’s in our cases were not felt to be pathologic; however, routine genetic analysis will enhance our ability to identify mutations of significance and help to shed light on the cause of cryptogenic cases.

Despite the importance of an early and thorough diagnostic workup, a recent literature review noted that only 60.9% of practitioners obtained autoimmune or paraneoplastic workup in NORSE/FIRES patients [5]. Investigation was limited to infectious or metabolic causes in 78% of studies. CSF cytokine measurements were obtained in as little as 21.4% of reports [47]. Urgent consultation with a pediatric rheumatologist and neurologist can aid in identifying what studies should be prioritized at the time of presentation. Most children who have their first seizure are taken to the closest ED for evaluation. Initial studies are often performed outside of a tertiary center where subspecialists are readily available for guidance. A high index of suspicion is needed by all providers when encountering a school-aged child that presents with their first unprovoked seizure so that the critical window for evaluation and treatment is not missed.

### 4.3. Proposed Treatment Guidelines

International consensus recommendations for the management of NORSE/FIRES were released in 2022, which include recommendations for initial treatment. It is highly recommended that systemic immunotherapy with IV steroids and/or IVIG be started within 72 h of onset of NORSE/FIRES [2]. These guidelines are modeled after consensus guidelines of the treatment of autoimmune encephalitis and other systemic autoimmune conditions that affect the CNS [48]. While response to initial SIT is poor in FIRES, it can be effective in cases where there is underlying autoimmune or paraneoplastic etiology. The incidence of an underlying autoimmune driven disorder is higher in adults than children. Most autoimmune workup will take longer than 72 h to result. Empiric treatment is recommended to avoid further neurologic damage, while diagnostic studies are pending [2]. Therapeutic plasma exchange (TPE) is another consideration as a first-line therapy. It has been favored less in pediatric cases likely due to increased immunosuppression and the risk of eliminating AEDs. It is highly effective and recommended in pediatric patients with autoantibody mediated disease [49]. In our cohort, first-line systemic immunomodulatory therapies were used within 48 h of RSE in all patients without improvement in seizure activity. TPE was used in all three patients as well; however, all three went on to require second-line therapies.

Consensus recommendations add that second-line SIT should be initiated within 7 days if first-line therapies are not effective. Specific recommendations on the type and dosage of second-line therapies are less clear. Adult practitioners within the consensus group favored rituximab followed by IL-6 antagonists, while pediatric clinicians preferred IL-1R antagonists followed by IL-6 antagonists [2]. It is recommended to consider rituximab if a pathogenic antibody is identified or suspected. The utilization of IL-1R antagonists or IL-6 antagonists should be considered in cryptogenic cases.

The evidence to support innate immune activation has made IL-1R antagonists a logical treatment for FIRES. Additionally, anakinra, an IL-1 receptor antagonist, readily crosses the blood–brain barrier, which would theoretically improve its activity against IL-1β produced by resident microglia and astrocytes. Despite the theoretical utility of IL-1R antagonism, its efficacy is variable [13,17,20,24,40,41,50]. A large review of second-line immunomodulation noted that anakinra was used in children with FIRES with seizure control or reduced seizure activity seen in 73% of patients in the acute phase [50]. This meta-analysis did not consistently report time to improvement in SE after starting anakinra, and most patients received other therapies. Neurologic outcomes were documented, and most patients continued to have post-NORSE epilepsy with moderate-to-severe disability occurring in 88% [50]. Our center’s experience with anakinra had less favorable results with two of the three requiring a switch to another cytokine-directed therapy before sedation could be discontinued. The one case that used IL-1β alone as a second-line therapy did have eventual control of seizures; however, it had the least favorable neurologic outcome. Due to complications of PRES and the prolonged duration of SE at presentation, it is difficult to determine if the utilization of another agent would have provided any benefit.

The use of tocilizumab, an IL-6 receptor antagonist, has resulted in favorable outcomes in many reports of NORSE/FIRES. It has been used more frequently in adults as compared with children in the current literature. When used in seven adults with NORSE (six cryptogenic, one autoimmune-mediated), SE was aborted in six of eight cases within ten days of administration. All patients showed elevated IL-6 levels in the CSF. Like the results with anakinra, five out of the six patients went on to have chronic seizure disorders at follow-up with severe-to-moderate disability in all but one [51]. Another retrospective analysis of adults with c-NORSE showed superior improvement in clinical outcome scores during follow-up when tocilizumab was used compared to those who received rituximab or no second-line therapy [38]. Again, many of these patients had elevated IL-6 in the CSF. Tocilizumab was used in two of our patients and led to the resolution of SE in one. CSF cytokines were normal in the former case and not obtained in the latter. Serum IL-6 was elevated early in the disease course in both patients. Subsequent analysis after starting tocilizumab showed higher levels of IL-6 in both patients and did not correlate with seizure burden. Tocilizumab blocks the IL-6 receptor and does not bind circulating IL-6, which makes post-initiation serum cytokine profiling less reliable [15,52]. The patient who had an abortion of seizures with tocilizumab was also the only patient who received rituximab; therefore, it is difficult to draw conclusions on which treatment provided the most benefit.

There is a paucity of the literature on the use of other systemic immunomodulatory therapies in cryptogenic NORSE/FIRES. JAKi proved to be efficacious in one of our patients who failed second-line therapies with rituximab, anakinra, and tocilizumab. One case report described a 5-year-old boy with FIRES treated effectively with emapalumab-Izsg, an IFN-γ neutralizing antibody, after failing treatment with anakinra, tocilizumab, and rituximab and subsequently developed secondary hemophagocytic lymphohistiocytosis (HLH). He was then transitioned to baricitinib, a first generation JAKi, without any chronic epilepsy syndrome and only mild cognitive impairment and ADHD [17]. A report of eight patients with autoimmune encephalitis refractory to initial immunotherapies were trialed on tofacitinib (JAKi), resulting in a good response in 25% and a partial response in 37.5%. In the two patients with a good response, one of them had NORSE and had seizure termination on day 16 of tofacitinib administration [16]. The JAK-STAT pathway has more recently been shown to be involved in the development of various forms of epilepsy [15]. The downstream effects of both type 1 interferons and IL-6 receptor activation is mediated through the JAK-STAT pathway. Additionally, JAKi are small molecule biologics and can cross the blood–brain barrier [53,54]. JAK inhibition may be a viable option for second-line treatment in FIRES cases resistant to initial immunomodulatory therapies. Baricitinib was used with success in seizure abortion in one of our patients after 48 h of use. It was used rather late in the clinical course, and, while seizure control was successful, significant neurologic deficits remained at the time of discharge in this case. IL-6 levels did show a more consistent decline after initiating treatment with baricitinib than with other immunomodulatory therapies in this case.

There are no current guidelines regarding the duration of SIT. A large retrospective cohort of c-NORSE in adults showed that clinical outcome scores at 2 years were best when SIT was continued beyond 18 weeks. The scores did not show meaningful improvement when therapy was continued beyond 52 weeks in this group [38]. The international consensus recommendations are to continue successful SIT for at least three months prior to discontinuation [2]. Immunomodulation has also been shown to be effective in the chronic phase of NORSE. One report of a 10-year-old child with FIRES who failed to improve on first-line SIT was given anakinra 1.5 years after disease onset and reached full seizure control for the first time in their disease course [41].

### 4.4. Outcomes

The outcomes of NORSE and FIRES in the pediatric population are ominous with an estimated 12% resulting in death [6,7,10,12,13,32,38,39]. Outcomes for survivors remain poor with 60–93% developing chronic epilepsy and neurocognitive impairment [7,13]. Poorer outcomes have been correlated with SRSE, longer times in a burst suppression coma, diffuse cortical edema and multifocal abnormalities on MRI [6,7,13]. In our cohort, all patients had significant neurologic impairment at the time of discharge. Two patients regained the ability to communicate at the time of discharge. The patient with the shortest time to seizure abortion and withdrawal of anesthetics had the best neurologic outcome at the time of discharge. One retrospective study looking at different cytokine profiles in patients with NORSE found that elevated innate immunity-associated inflammatory cytokines were associated with worse outcomes at discharge [39]. The utilization of second-line therapy has resulted in improved outcomes when compared to those who only receive IVIG and corticosteroids [38]. Another literature review focused on the efficacy of second-line immunotherapies, specifically anakinra, tocilizumab, and intrathecal dexamethasone. While seizure burden improved in most reports, patients continued to experience long-term neurologic disabilities [50]. Outcomes are often reported as poor at the time of discharge; however, nearly half of patients continue to experience neurologic improvements over time [10]. The international consensus recommendations highlight the importance of neuropsychological evaluation upon discharge in all patients with NORSE/FIRES with the implementation of individualized therapies. Psychiatric evaluation is also important as mood disorders are common. Intensive motor and cognitive rehabilitation is essential for optimizing neurologic outcomes [2].

## 5. Conclusions

FIRES is a catastrophic condition characterized by the abrupt onset of SE in previously healthy individuals. The aberrant activation of the innate immune system is postulated as part of the pathogenesis of FIRES prompting the utilization of immunomodulatory therapies as the standard of care. Second-line immunomodulatory therapies should be considered early when patients do not respond to first-line treatment. A thorough and comprehensive evaluation prior to initiating treatment is crucial in identifying which cases are likely to be cryptogenic. Our cohort highlights that, despite similarities in initial presentation and early uses of IVIG and glucocorticoids, second-line therapies were required in all cases. It also highlights the difficulty in deciding which second-line therapy will provide the most benefit based on individual patient characteristics. While all three patients showed innate immune activation by serum cytokine profile, only two patients had cytokine analysis early in the disease course. Cytokines were not analyzed prior to initiation of SIT in any case making results difficult to interpret. Neurocognitive deficits were seen in varying degrees of severity in all patients despite each receiving first-line SIT within the first 48 h. Poorly controlled and prolonged seizures resulted in the most severe neurologic outcomes. Each patient received several second-line immunomodulatory therapies with multiple infections during their treatment. A more targeted approach to treatment may decrease the burden of immunosuppression; however, targeted therapy can only be provided if the initial workup is thorough. The consideration of JAKi in the treatment of FIRES may allow for more robust cytokine-directed therapy in refractory cases. Further research is needed to identify different genetic or immunologic susceptibilities in FIRES as well as to improve upon treatment and outcomes. Prospective studies are needed to standardize autoimmune analysis and cytokine profiling at the time of presentation in both serum and the CSF. Repeat cytokine analysis at structured timepoints can help clarify correlations with treatment modalities and clinical findings. Implementing second-line therapies earlier in the clinical course, when data suggest c-FIRES, may help lessen time to seizure control and improve outcomes. Further data are needed to clarify how long to trial a specific cytokine-directed therapy is before deeming it ineffective. Routine genetic analysis on FIRES patients is essential in efforts to understand the interplay between abnormalities in immune regulation and seizure development.

## Figures and Tables

**Table 2:** Clinical characteristics of three FIRES patients.

	Case 1	Case 2	Case 3
Age in Years/Sex	10/M	6/M	8/F
Symptoms prior to SE (days)	Headache (6) Fever (6)	Fever (6) Fatigue (6) Anorexia (6) Chest pain (0)	Sore throat (6) Abdominal pain (6) Fever (4) Anorexia (4) Fatigue (0) Headache (0)
Family history of autoimmune disease	Sister with juvenile idiopathic arthritis	None	Father with history of juvenile idiopathic arthritis
EEG findings	Background: Generalized polymorphic 2–4 Hz slowing. Seizure: Excessive beta frequencies. Right temporoparietooccipital seizures and focal seizure with secondary generalized seizures.	Background: Diffuse high-amplitude delta slowing. Seizures: Occasional small spikes from the left temporal central area or right central parietal area, which progressed to generalized seizures.	Background: High-amplitude delta slowing (2–3 Hz) with intermittent frontal central spindles. Seizures: Originating in the left centrotemporal region that spread to the contralateral hemisphere. Evolved into seizures with diffuse onset.
MRI findings	Initial: Symmetric restricted diffusion in the hippocampus Repeat: Bilateral hippocampal volume loss and diffuse generalized cerebral volume loss.	Initial: Symmetric T2/FLAIR hyperintensities in the bilateral basal ganglia and right nuclei. Repeat after prolonged SE: Cytotoxic edema with scattered petechial hemorrhages and vasogenic edema in bilateral parietooccipital parenchyma and posterior cingulate gyri (hemorrhagic PRES).	Initial: FLAIR hyperintensity of the sulci.
CSF	Initial: RBC 95, glucose 84 Repeat: RBC 420, glucose 91 MBP 6.11	Initial: Glucose 106 Repeat: RBC 2000, protein 82 IgG synthesis 7.9 (N 0–2.2 mg/24 h) MBP 29.4	Initial: neutrophil 53%, glucose 85% Repeat: RBC 124
Autoantibodies	CSF: GAD 65: 0.09 (N < 0.02 nmol/L) Serum: Anti-thyroglobulin ab 2.4 (N < 0.4 U/mL) TPO 52.2 (N < 20 IU/mL) GAD 65: 44.9 (N < 5 IU/mL) DRVVT positive SSA 21 (N < 20 U)	CSF: GAD 65: 0.05 (N < 0.02 nmol/L) Serum: Anti-thyroglobulin ab 0.7 (N < 0.4 U/mL)	CNS: GAD 65: 0.16 (N < 0.02 mmol/L) Serum: SSA 50 (N < 20 U)
Genetics	VUS in *KIF1A*	VUS in *KCNH2* and *MICAL1*	Not obtained
Antiepileptics used in sequence	Levetiracetam Lacosamide Fosphenytoin Perampanel Clobazam Brivaracetam Cenobamate Felbamate Ganloxone	Levetiracetam Valproate Fosphenytoin Lacosamide Perampanel Epidiolex Diazepam Phenobarbital Cenobamate Felbamate Topiramate	Levetiracetam Fospheytoin Lacosamide Valproate Brivaracetam Clobazam Phenobarbital Cenobamate Cannabidiol Perampanel
Continuous IV anesthetics (duration in days)	Midazolam (4) Pentobarbital (14) Ketamine (58)	Midazolam (6) Pentobarbital (42) Ketamine (85) Propofol (13) Dexmedetomidine (22)	Midazolam (17) Pentobarbital (24) Dexmedetomidine (6)
AEDs on discharge	Phenobarbital Cenobamate Felbamate Brivaracetam Lacosamide Clobazam Perampanel	Levetiracetam Lacosamide Diazepam Phenobarbital Cenobamate Topiramate Perampanel	Lacosamide Clobazam Phenobarbital Cenobamate Cannabidiol Divalproex Perampanel
Adverse events	Tracheitis; ESBL UTI; ventilator-acquired pneumonia; bacteremia; drug-induced liver injury	PRES; drug-induced liver injury; ESBL UTI; bilateral renal calculi	Hypogammaglobulinemia; acute severe macroglossia; renal calculi; thrombocytopenia; UTI; pneumonia

Cerebral spinal fluid (CSF); normal CSF glucose (43–73 mg/dL); normal CSF protein (15–45 mg/dL); normal CSF WBC (0–5/cm); normal CSF seg neutrophils (0–6%); normal CSF RBC (0–10/cm); myelin basic protein (MBP) 0.00–5.50 ng/mL; variant of uncertain significance (VUS); posterior reversible encephalopathy syndrome (PRES); extended-spectrum beta lactamase (ESBL); and urinary tract infection (UTI).

**Table 3:** Systemic immunotherapy used in three FIRES patients.

SIT	Case 1	Case 2	Case 3
IV methylprednisolone (doses/days)	1 g × 3 (9 doses)	10 mg/kg BID (5 days) 20 mg/kg (1 dose) 30 mg/kg (2 doses)	40 mg/kg (5 days)
IVIG	2 g/kg over 4 days	2 g/kg over 2 days (2 doses)	2 g/kg over 3 days
Plasmapheresis	5 days	5 days on alternating days	5 days
Anakinra	4 mg/kg/day (9 days) 8 mg/kg/day (12 days)	5 mg/kg sq Q 12 (15 days) 2–4 mg/kg/day IV *	4 mg/kg/day increased to 10 mg/kg/day
Rituximab			750 mg/m^2^ (2 doses)
Tocilizumab	8 mg/kg × 1		8 mg/kg every 14 days *
Baricitinib	4 mg daily 2 mg daily *		

* Systemic immunotherapy continued at time of discharge

**Table 4:** Elevated cytokine levels over time.

Case 1	DOH: 8 Seizure: SE	DOH: 23; 28 Seizure: Subclinical Seizures	DOH: 50 Seizures: Subclinical Focal Seizures	DOH: 63 Seizures: None Weaning Sedation	DOH: 91 Seizures: 1 Subclinical Seizure. Off Sedation
SIT to date	methylprednisolone, IVIG	anakinra, plasmapheresis, IVIG, tocilizumab	no active SIT	baricitinib	baricitinib
Cytokines	Serum: * IL-6: 71 pg/mL * IL-8: 31 pg/mL	Serum ^+^ IL-6: 20.5 pg/mL; 12.4 pg/mL	Serum: * IL-6: 71 pg/mL ** CSF: Normal	Serum: * IL-6: 157 pg/mL * IL-8: 43 pg/mL	Serum: ^+^ IL-6: 4.6 pg/mL
Case 2	DOH: 38 Seizures: SE		DOH: 56 Seizures: Focal		DOH: 102 Seizure: None
SIT to date	methylprednisolone IVIG plasmapheresis anakinra		anakinra		methylprednisolone anakinra
Cytokines	CSF: ** IL-6: 71 pg/mL		Serum: ^ IL-2Rs: 2981 pg/mL ^+^ IL-6: 3.8 pg/mL		Serum: * IL-8: 26 pg/mL * IFN gamma: 2 pg/mL ** CSF: normal
Case 3	DOH: 6 Seizures: SE		DOH: 13 Seizures: Self-limited seizures		DOH: 40 Seizures: None
SIT to date	methylprednisolone IVIG		plasmapheresis anakinra		rituximab tocilizumab
Cytokines	Serum: ^+^ IL-6: 8.6 pg/mL ^+^ IL-8: 5.0 pg/mL		Serum: ^+^ IL-2 R soluble: 1308.6 pg/mL ^+^ IL-5: 9.5 pg/mL ^+^ IL-10: 10.9 pg/mL ^+^ IL-18: 1.8 pg/mL ^+^ IL-6: 21.7 pg/mL ^+^ IL-8: 3.5 pg/mL		Serum: ^+^ IL-2 Rs: 2071.2 pg/mL ^+^ IL-10: 7.8 pg/mL ^+^ IL-6: 37.8 pg/mL

SIT = systemic immune therapy; DOH = day of hospitalization. Cytokine analysis: * Cincinnati Children’s Laboratory Serum: IL-1 beta ≤ 4 pg/mL, IL-2 ≤ 8 pg/mL, IL-4 ≤ 19 pg/mL, IL-5 ≤ 5 pg/mL, IL-6 ≤ 8 pg/mL, IL-8 ≤ 9 pg/mL, IL-10 ≤ 7 pg/mL, IFN gamma ≤ 1 pg/mL, TNF alpha ≤ 3 pg/mL, GM-CSF ≤ 4 pg/mL; ** Cincinnati Children’s Laboratory CSF: IL-1 beta ≤ 4 pg/mL, IL-2 ≤ 6 pg/mL, IL-4 ≤ 16 pg/mL, IL-5 ≤ 5 pg/mL, IL-6 ≤ 63 pg/mL, IL-8 ≤ 181 pg/mL, IL-10 ≤ 6 pg/mL, IFN gamma ≤ 1 pg/mL, TNF alpha = 3 pg/mL, GM-CSF ≤ 3 pg/mL; ^+^ ARUP Laboratories Serum: IL-2 ≤ 2.1 pg/mL, IL-2 Rs 175.3–858.2 pg/mL, IL-12 ≤ 1.9 pg/mL, IFN gamma ≤ 4.2 pg/mL, IL-4 ≤ 2.2 pg/mL, IL-5 ≤ 2.1 pg/mL, IL-10 ≤ 2.8 pg/mL, IL-13 ≤ 2.3 pg/mL, IL-17 ≤ 1.4 pg/mL, IL-1 B ≤ 6.7 pg/mL, IL-6 ≤ 2 pg/mL, IL-8 ≤ 3 pg/mL, TNF-a ≤ 7.2 pg/mL; and ^ Quest Diagnostics Serum: IL-2 Rs 532–1891 pg/mL.

## Data Availability

The original contributions presented in this study are included in this article. Further inquiries can be directed to the corresponding author.

## Abbreviations

The following abbreviations are used in this manuscript:

ADEM	Acute demyelinating encephalomyelitis
ADHD	Attention-deficit hyperactivity disorder
AEDs	Anti-epileptic drugs
ANA	Anti-nuclear antibody
ANCA	Anti-neutrophil cytoplasmic antibody
Anti-TPO	Anti-thyroid peroxidase
BBB	Blood–brain barrier
BID	Twice a day
c-FIRES	Cryptogenic FIRES
c-NORSE	Cryptogenic NORSE
CRP	C-reactive protein
CSF	Cerebral spinal fluid
CT	Computed tomography
DOAJ	Directory of open access journal
DRVVT	Dilute Russell’s viper venom time
ED	Emergency department
EDB	Extreme delta brush
EEG	Electroencephalogram
FIRES	Febrile infection-related epilepsy syndrome
FLAIR	Fluid-attenuated inversion recovery
GAD	Glutamic acid decarboxylase
HLH	Hemophagocytic lymphohistiocytosis
HSV	Herpes simplex virus
IFN	Interferon
IgG	Immunoglobulin G
IL-1R	Interleukin 1 receptor
IL-2Rα	Interleukin 2 receptor alpha
IVIG	Intravenous immunoglobulin
JAKi	Janus kinase inhibitor
MBP	Myelin basic protein
MDPI	Multidisciplinary Digital Publishing Institute
MOG	Myelin oligodendrocyte glycoprotein
MRA	Magnetic resonance angiography
MRI	Magnetic resonance imaging
MRV	Magnetic resonance venography
NMDA	N-methyl-D-aspartic acid
NORSE	New onset refractory status epilepticus
PICU	Pediatric intensive care unit
PRES	Posterior reversible encephalopathy syndrome
RBCs	Red blood cells
RSE	Refractory status epilepticus
SE	Status epilepticus
SIT	Systemic immunotherapy
SLE	Systemic lupus erythematosus
Sq	subcutaneous
SRSE	Super refractory status epilepticus
SSA	Anti-Sjogren’s-syndrome-related antigen A
TLR	Toll-like receptor
TNF-α	Tumor necrosis factor alpha
TPE	Therapeutic plasma exchange
VUS	Variant of uncertain significance
WBC	White blood cell
